# Antibiotic and Nonantibiotic Drugs Associated With *Clostridioides difficile* Infection Risk: a Pharmacopoeia-Wide Case-Cohort Study

**DOI:** 10.1093/infdis/jiag001

**Published:** 2026-03-16

**Authors:** Kevin A Brown, Bryan Coburn, Alejandro Hernandez, Bradley J Langford, Valerie Leung, Derek MacFadden, Ashley M Rooney, Kevin L Schwartz, Nick Daneman

**Affiliations:** Public Health Ontario, Toronto, Ontario, Canada; Dalla Lana School of Public Health, University of Toronto, Toronto, Ontario, Canada; ICES, Toronto, Ontario, Canada; Department of Medicine, University of Toronto, Toronto, Ontario, Canada; Toronto General Hospital Research Institute, Toronto, Ontario, Canada; ICES, Toronto, Ontario, Canada; Public Health Ontario, Toronto, Ontario, Canada; Dalla Lana School of Public Health, University of Toronto, Toronto, Ontario, Canada; Public Health Ontario, Toronto, Ontario, Canada; Ottawa Hospital Research Institute, Ottawa, Ontario, Canada; Department of Medicine, University of Ottawa, Ottawa, Ontario, Canada; Institute of Medical Microbiology, University of Zurich, Zurich, Switzerland; Public Health Ontario, Toronto, Ontario, Canada; Dalla Lana School of Public Health, University of Toronto, Toronto, Ontario, Canada; ICES, Toronto, Ontario, Canada; Public Health Ontario, Toronto, Ontario, Canada; ICES, Toronto, Ontario, Canada; Division of Infectious Diseases, Sunnybrook Research Institute, Toronto, Ontario, Canada; Institute of Health Policy, Management and Evaluation, University of Toronto, Toronto, Ontario, Canada

**Keywords:** *Clostridium difficile*, antibiotic-associated diarrhea, microbiome, drug surveillance

## Abstract

**Background:**

*Clostridioides difficile* infection (CDI) is principally precipitated by antibiotics, due to their disruption of gut commensal bacteria. The comparative role of nonantibiotic drugs is poorly characterized.

**Methods:**

We examined the contribution of antibiotic and nonantibiotic drugs to CDI risk among residents age >65 years old and not hospitalized in the prior 30 days, between 2018 and 2023. The study used a case-cohort study design, with logistic regression analysis. The case definition consisted of first incident CDI, identified using comprehensive *C. difficile* testing, hospitalization, and treatment data. Outpatient oral drug exposures were measured in a 1–90-day window prior to case and control days. Adjusted regression models included covariates for age, sex, year and quarter, region, comorbid conditions, healthcare exposures, and drug exposures.

**Results:**

Among 16 196 CDI case patients and 549 831 controls, 335 drugs were included. After adjustment, the antibiotics amoxicillin-clavulanate (odds ratio [OR], 6.05 [95% confidence interval (CI), 5.69–6.43]), clindamycin (16.83 [15.53–18.24]), ciprofloxacin (3.83 [3.59–4.09]), and cephalexin (3.05 [2.86–3.25]), were the largest contributors to CDI risk. Nonantibiotic drugs pantoprazole (OR, 1.33 [95% CI, 1.27–1.39]) and ferrous fumarate (1.71 [1.61–1.82]) were the next largest. Metformin had a protective association (OR, 0.67 [95% CI, .63–.72]). In a meta-regression on a subset of 182 drugs, in vitro anticommensal activity was positively associated with CDI risk (*P* < .001).

**Conclusions:**

This study provides insights into CDI etiology and avenues for stewardship and drug repurposing to combat CDI and antimicrobial resistance.

## Graphical Abstract

**Graphical Abstract:**
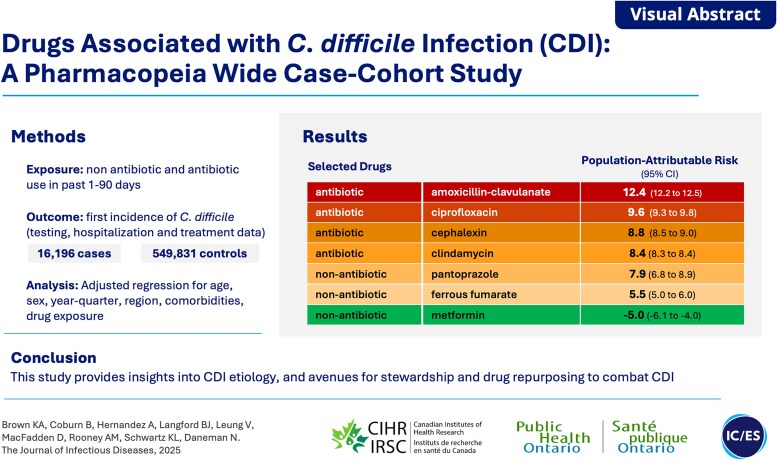

## Teaser

We estimated the contribution of antibiotic and nonantibiotic drugs to *Clostridioides difficile* infection risk over a 6-year period. Antibiotics including amoxicillin-clavulanate, clindamycin, ciprofloxacin, and cephalexin generated the most risk, though nonantibiotics, particularly pantoprazole and ferrous fumarate, also contributed.

**(See the Editorial Commentary by Halpin and McDonald on pages e1088–91.)**

*Clostridioides difficile* is an antimicrobial resistant bacterium that can cause infection in the gastrointestinal tract, leading to diarrhea, pseudomembranous colitis, and severe outcomes, including death. Globally, *C. difficile* infection (CDI) causes a loss of 2.1 million adjusted life-years annually [1], and there are >400 000 cases annually in the United States [2]. CDI is principally precipitated by exposure to antibiotics, in particular 4C antibiotics (clindamycin, amoxicillin-clavulanate, cephalosporins, and ciprofloxacin/fluoroquinolones). One hypothesis regarding why some antibiotics cause greater risk than others is related to their spectrum of antimicrobial activity; anti-anaerobic activity is thought to increase risk, while anti–*C. difficile* activity is thought to decrease risk [3]. In addition to antibiotics, a large number of nonantibiotic drugs are known to have substantial antimicrobial activity and as such could cause CDI [4].

However, there is little comprehensive information on the total contribution of antibiotic and nonantibiotic drug exposures to the overall burden of CDI. We sought to measure associations between all prescribed oral drug exposures and CDI risk. We further sought to measure whether CDI risk could be predicted based on the in vitro antimicrobial activity of drugs against gut commensal bacteria [4].

## METHODS

### Case and Control Selection

We developed a longitudinal cohort of CDI case patients and population-representative controls in the period from 1 January 2018 to 31 December 2023. All data were accessed through ICES, an independent, nonprofit research institute. The case definition consisted of the first incident CDI, defined as a positive result of a laboratory *C. difficile* toxin test (enzyme immunoassay or a nucleic acid amplification test) using unformed stool, hospitalization with CDI based on *International Classification of Diseases, Tenth Revision* code A04.7, or prescription for oral vancomycin or fidaxomicin (drugs used for CDI treatment [5]), whichever came first. We used a case-cohort design, and we enumerated every at-risk person-day up to the first incident CDI, death, or the end of follow-up, whichever came first. Next, days on which persons experienced the outcome were considered case patients; controls consisted of a 0.01% simple random sample of days on which persons in the study population did not experience the outcome. Some individuals could be included as both controls and subsequently as case patients. This unmatched approach combines the benefits of the matched nested case-control design (efficient and longitudinal) with those of an unmatched cohort design (representativity) [6].

### Inclusion and Exclusion Criteria

Case patients and controls ≥66 years of age were eligible for inclusion. We excluded persons <66 years of age since drug receipt information was sourced from a public drug funding program (Ontario Drug Benefit) covering all Ontarians aged ≥65 years. An additional year of age was excluded to enable retrospective capture of drug receipt. We excluded persons after their first instance of CDI during the study period. For our primary analysis, we excluded case and control days with a hospital exposure in the prior 3–30 days; this meant we could include case patients or controls during the initial 2 days of a hospitalization. For our secondary analysis, we excluded case patients and controls with a hospital exposure in the prior 1–90 days.

### Exposure

Receipt of oral drugs was captured via the database of the Ontario Drug Benefit program. The database records individual drug dispensing records for >5000 covered drugs, with an error rate of <1% [7]. Drugs that are prescribed but not dispensed are not captured. Drugs were labeled in accordance with their anatomic therapeutic classification (ATC) name. We further classified drugs according to level 2 and 4 of their ATC code [8]. For example, the full ATC code for pantoprazole is A02BC02; the level 2 code is A02 (drugs for acid-related disorders), and the level 4 code is A02BC (proton pump inhibitors [PPIs]). For each case patient and control, we searched for receipt of each drug in a retrospective window. Any receipt within the window was binarily classified based on dispensing date and the number of days a drug was supplied. We included any drug received by ≥100 controls. Our primary analysis was based on a 1–90-day retrospective exposure window. For our secondary analysis, we used a 7–90-day retrospective exposure window to better mitigate the effects of drugs that may be used to treat CDI symptoms and would be subject to protopathic (reverse causation) bias in the primary 1–90-day exposure window [9]. As additional sensitivity analyses (SAs), we measured exposures in a 1–30-day retrospective window to assess short-duration associations, and in a 1–180-day retrospective window to assess for long-duration associations.

### Covariates

We included the following covariates: age (66–69, 70–74, 75–79, 80–84, 85–89, or ≥90 years), sex (male or female), region (eastern, central, metro Toronto, southwestern, or northern), year and quarter (ie, 2018 quarter 1 through 2023 quarter 4), time in hospital in the prior year (0, 1–2, 3–6, 7–13, 14–27, and ≥28 days), time in the intensive care unit in the prior year (0, 1–6, or ≥7 days), and stay in a long-term care home in prior year (yes or no). We also included comorbid conditions as risk factors, including inflammatory bowel disease and each of the 17 components of the Charlson comorbidity score (myocardial infarction, congestive heart failure, peripheral vascular disease, cerebrovascular disease, dementia, chronic obstructive pulmonary disease, rheumatic disease, peptic ulcer disease, mild liver disease, moderate or severe liver disease, diabetes mellitus without or with complications, hemiplegia or paraplegia, renal disease, primary cancer, metastatic cancer, and human immunodeficiency virus/AIDS).

### Statistical Analyses

Analyses used logistic regression models treating case versus control status as a binary outcome variable. Unadjusted and adjusted models were fitted. Unadjusted models consisted of a separate model for each drug exposure and included that drug as the only variable. Adjusted models included variables for all eligible drugs, in addition to all listed covariates (age, sex, year and quarter, region, healthcare exposures and comorbid conditions). We further measured the population-attributable risk (PAR) of CDI for each drug, based on the prevalence of drug exposure among case patients, multiplied by the attributable risk among case patients (1 − 1/odds ratio [OR]) [10]. The PAR provides an estimate of the proportionate reduction in cases if the exposure was removed from the population. Negative values signified a proportionate increase if the exposure was removed. We summed antibiotic and nonantibiotic drug-associated PARs to get an estimate of the portion of drug-associated PAR attributable to antibiotics versus nonantibiotics. *P* values corresponding to each OR were Bonferroni corrected by multiplying their *P* value by the number of drugs tested.

A total of 7 pharmacopeia-wide analyses were performed. The primary analysis was adjusted and used a 1–90-day retrospective drug exposure window and a 3–30-day retrospective hospitalization exclusion window. The secondary analysis used a robust 7–90-day drug window, to protect against potential reverse causation for drugs that may have been initiated as treatments for CDI symptoms but prior to diagnosis, and a more stringent 1–90-day hospitalization exclusion, to protect against drug exposure misclassification since drug exposures were not captured during hospitalization.

The 5 SAs represented single modifications to the primary analysis. The 7 analyses are summarized as follows: primary analysis (adjusted; 1–90-day drug exposure and 3–30-day hospital exclusion); secondary analysis (adjusted; 7–90-day drug exposure and 1–90-day hospital exclusion); SA1—unadjusted (unadjusted; 1–90-day drug exposure and 3–30-day hospital exclusion); SA2—short drug exposure (adjusted; 1–30-day drug exposure and 3–30-day hospital exclusion); SA3—long drug exposure (adjusted; 1–180-day drug exposure and 3–30-day hospital exclusion); SA4—robust drug exposure (adjusted; 7–90-day drug exposure and 3–30-day hospital exclusion); and SA5—long hospitalization exclusion (adjusted; 1–90-day drug exposure and 1–90-day hospital exclusion). To systematically compare drug association estimates from primary and secondary analyses and 5 SAs, we examined linear associations between the primary analysis and each of the 6 other analyses; for each, we identified the top 5 most different estimates based on the log OR.

For all drugs with existing data on antimicrobial activity against a panel of 40 gut bacteria (Supplementary Table 1 [4]), we conducted a meta-regression to examine potential mechanisms underlying observed drug effects. For these analyses the outcome was the adjusted drug log OR from the primary and secondary analyses. We included 2 exposure variables: (1) the antimicrobial activity of the drug against *C. difficile* and (2) the mean of the antimicrobial activity against 39 gut commensal bacteria. Maier et al [4] reported *P* values as measures of antimicrobial activity, where values close to 1 indicated no activity, and values close to 0 represented high activity. These *P* values were converted to *S* values by rescaling as −log_10_ (*P* value).

Activity was on a continuous scale from 0 to 6.6, where 0 indicated no activity (corresponding to a *P* value of 1) and 6.6 indicated high activity (corresponding to the minimum recorded *P* value of 2.7 × 10^−7^) [11]. For anti–*C. difficile* activity, this was the only step required, while for mean commensal activity, we subsequently took the average of the 39 antimicrobial activity values. Because certain subgroups of gut commensal bacteria may be hypothesized to be particularly protective against CDI, including anaerobes, bile salt hydrolases and butyrate producers [12, 13], we also conducted analyses replacing mean anticommensal activity with activity against anaerobic bacteria (29 bacteria), butyrate producers (7 bacteria [14]), and bile salt hydrolase inhibitors (20 bacteria [15]). We hypothesized that increased anti–*C. difficile* activity would be associated with decreased risk and that increased anti–gut commensal activity would be associated with increased risk.

Logistic regression models were fitted with SAS 9.4 software, while meta-regression models were fitted with R 4.4.0 software using the metafor 4.6-0 package [16].

### Ethics

This study was approved by the research ethics board of Public Health Ontario.

## RESULTS

### Characteristics

**Figure 1.**
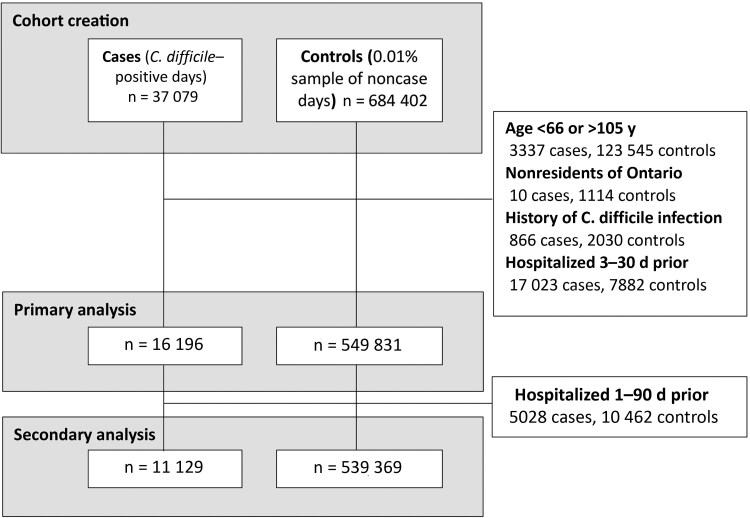
Flow chart describing the selection of case patients and controls in the case-cohort study design. Case patients represented days on which persons developed *Clostridioides difficile* infection. Controls represented a 0.01% sample (1 in 10 000) of noncase days. In a case-cohort design, controls can subsequently become case patients.

We identified 16 196 CDI case patients and 549 831 population-representative controls in the primary analysis (Figure 1). Case patients, compared with controls, were more likely to be female (60.2% vs 54.4% [standardized mean difference, 0.12), older (aged ≥90 years; 11.0% vs 5.7% [0.19]), have a history of recent prolonged hospitalization (≥28 days; 9.0% vs 0.6% [0.40]), and have comorbid conditions, particularly diabetes mellitus with complications (13.3% vs 2.4% [0.41]), congestive heart failure (9.1% vs 1.4% [0.35]), renal disease (5.4% vs 0.6% [0.29]), and cancer (6.6% vs 1.3% [0.28]) (Table 1). The secondary analysis, excluding hospital admissions in the prior 90 days, yielded 11 129 case patients and 539 369 controls (Figure 1).

**Table 1.** Characteristics of Case Patients and Controls in the Primary Analysis

Characteristics	Case Patients, No. (%) (n = 16 196)	Controls, No. (%) (n = 549 831)	SMD
Female sex	9744 (60.2)	192 097 (54.4)	0.116
Age group, y			
66–69	2633 (16.3)	143 915 (26.2)	0.243
70–74	3499 (21.7)	150 432 (27.4)	0.133
75–79	3187 (19.7)	106 228 (19.3)	0.01
80–84	2767 (17.1)	72 201 (13.1)	0.112
85–89	2297 (14.2)	45 463 (8.3)	0.189
≥90	1774 (11.0)	31 592 (5.7)	0.19
Time spent in acute care, d			
0	8320 (51.5)	503 451 (91.6)	0.991
1–2	2086 (12.9)	14 183 (2.6)	0.394
3–6	1455 (9.0)	14 241 (2.6)	0.277
7–13	1503 (9.3)	9044 (1.6)	0.342
14–27	1339 (8.3)	5341 (1.0)	0.354
≥28	1454 (9.0)	3571 (0.6)	0.397
Time spent in ICU, d			
0	14 972 (92.7)	543 441 (98.8)	0.31
1–2	485 (3.0)	3269 (0.6)	0.182
3–6	364 (2.3)	2151 (0.4)	0.164
≥7	336 (2.1)	970 (0.2)	0.181
Long-term care in prior year			
No	15 039 (93.1)	538 746 (98.0)	0.239
Yes	1118 (6.9)	11 085 (2.0)	0.239
Comorbid conditions			
IBD	168 (1.0)	287 (0.1)	0.134
MI	533 (3.3)	5067 (0.9)	0.166
CHF	1470 (9.1)	7950 (1.4)	0.348
PVD	443 (2.7)	2503 (0.5)	0.183
CVD	560 (3.5)	5092 (0.9)	0.174
Dementia	708 (4.4)	5175 (0.9)	0.215
COPD	950 (5.9)	6100 (1.1)	0.262
Connective tissue/rheumatic disease	144 (0.9)	682 (0.1)	0.108
Peptic ulcer disease	233 (1.4)	1123 (0.2)	0.137
Mild liver disease	176 (1.1)	669 (0.1)	0.125
Moderate or severe liver disease	82 (0.5)	284 (0.1)	0.086
DM without complications	644 (4.0)	7820 (1.4)	0.159
DM with complications	2143 (13.3)	13 275 (2.4)	0.412
Hemiplegia or paraplegia	162 (1.0)	880 (0.2)	0.111
Renal disease	876 (5.4)	3100 (0.6)	0.288
Primary cancer	1069 (6.6)	7143 (1.3)	0.275
Metastatic cancer	437 (2.7)	1832 (0.3)	0.195
HIV-AIDS	6 (0.0)	20 (0.0)	0.023
Region			…
Eastern	2643 (16.4)	94 779 (17.2)	0.024
Central	6482 (40.1)	202 523 (36.8)	0.068
Metropolitan Toronto	2319 (14.4)	104 310 (19.0)	0.124
Southwestern	3143 (19.5)	109 540 (19.9)	0.012
Northern	1570 (9.7)	38 679 (7.0)	0.097

Abbreviations: CHF, congestive heart failure; COPD, chronic obstructive pulmonary disease; CVD, cerebrovascular disease; DM, diabetes mellitus; HIV, human immunodeficiency virus; IBD, inflammatory bowel disease; ICU, intensive care unit; MI, myocardial infarction; PVD, peripheral vascular disease; SMD, standardized mean difference.

### Drugs

For the primary analysis (adjusted, with a 1–90 drug exposure window and a 3–30-day hospitalization exclusion), we identified 335 drugs meeting our criterion of being received on ≥100 control days. The most commonly prescribed drug classes among controls were lipid-modifying agents, antihypertensive drugs acting on the renin–angiotensin–aldosterone system, and drugs used for diabetes mellitus. The most commonly prescribed drug classes among case patients were antibiotics, lipid-modifying agents, and analgesics. For the secondary analysis (adjusted, with a 7–90-day exposure window and a 1–90-day hospitalization exclusion), 330 of 335 drugs from the primary analysis (98.5%) met the inclusion criteria.

### Drug CDI Risk

**Figure 2.**
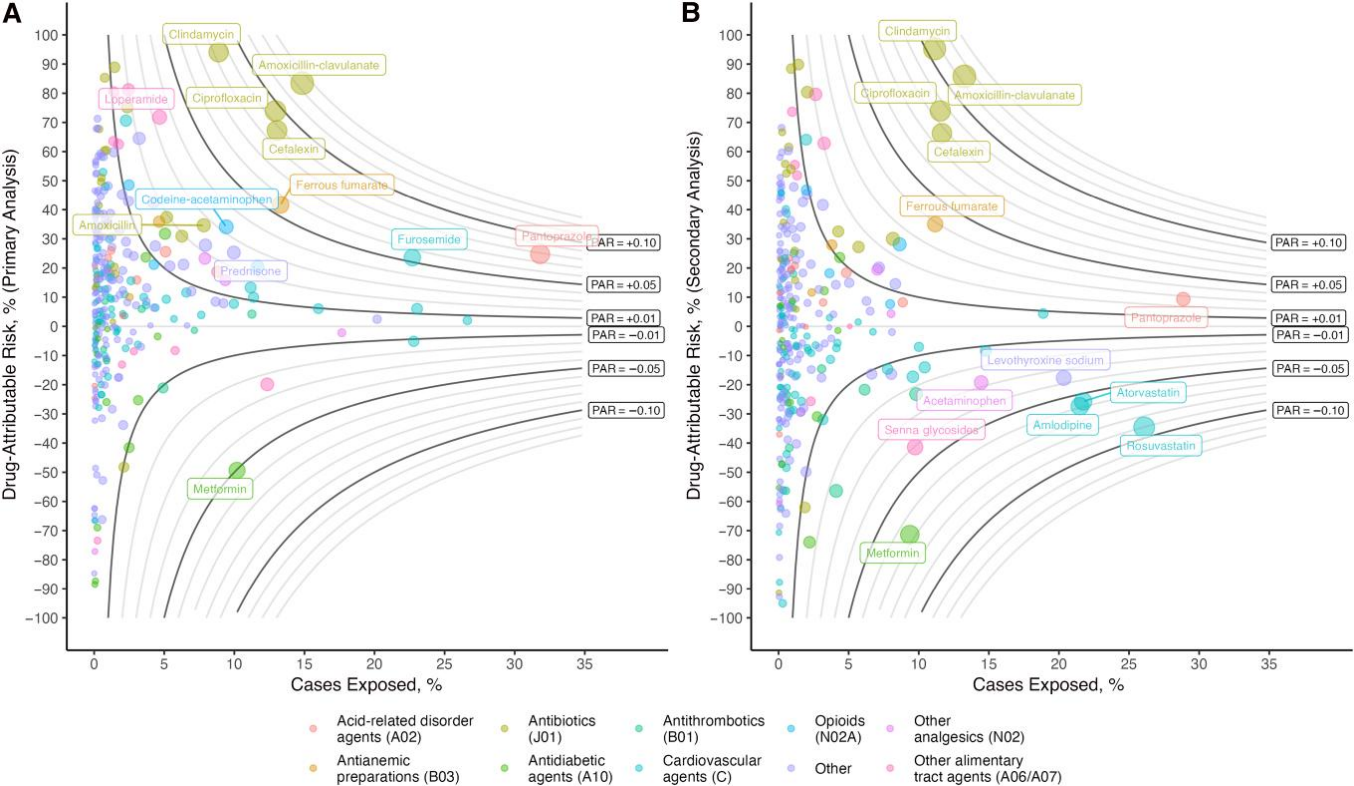
Population-attributable risk (PAR) of *Clostridioides difficile* infection (CDI) for drugs prescribed in Ontario, based on primary (*A*; n = 335), and secondary (*B*; n = 330) analyses. The y-axes show the drug-attributable risk (1 − 1/odds ratio [OR]) × 100%), while the x-axes show the proportion of cases exposed. Nonlinear lines represent the PAR, equal to the attributable risk multiplied by the proportion of cases exposed. A positive PAR denotes the estimated percentage decrease in CDI incidence that would be expected from removal of the given drug exposure (among drugs with ORs >1), while a negative value indicates the percentage increase in incidence expected (among drugs with ORs <1).

In the primary analysis, 19% of drugs (64 of 335) had a statistically significant positive OR association (OR >1) with CDI risk, while 2% (8 of 335) had a significant negative association (OR <1; Figure 2). We evaluated the percentage of cases attributable to each drug exposure in our population (PAR); 15 drugs had a PAR of >2%, while 2 had a PAR of <−2% (Table 2). In the secondary analysis (1–90-day hospitalization exclusion and 7–90-day drug exposure window), 11% of drugs (36 of 330) had a significant positive association, while 5% (17 of 330) had a significant negative association. Ten drugs had a PAR >2%, while 9 had a PAR <− 2%.

**Table 2.** **Drugs With Population-Attributable Risk >2% or <−2% in the Primary and Secondary Analyses** ^a^

Drug	ATC	Primary Analysis (16 196 Case Patients and 549 831 Controls)					Secondary Analysis (11 129 Case Patients and 539 369 Controls)				
		No. (%)		Adjusted OR (95% CI)	PAR (95% CI)	*P* Value^b^	No. (%)		Adjusted OR (95% CI)	PAR (95% CI)	*P* Value^b^
		Case Patients	Controls				Case Patients	Controls			
PAR ≥2%											
Amoxicillin-clavulanate	J01CR02	2404 (14.8)	7559 (1.4)	6.05 (5.69–6.43)	12.4 (12.2–12.5)	<.001	1475 (13.3)	6249 (1.2)	7.02 (6.53–7.56)	11.4 (11.2–11.5)	<.001
Clindamycin	J01FF01	1438 (8.9)	2759 (0.5)	16.8 (15.5–18.2)	8.4 (8.3–8.4)	<.001	1239 (11.1)	2490 (0.5)	20.9 (19.2–22.8)	10.6 (10.6–10.6)	<.001
Ciprofloxacin	J01MA02	2095 (12.9)	8154 (1.5)	3.83 (3.59–4.09)	9.6 (9.3–9.8)	<.001	1284 (11.5)	6960 (1.3)	3.84 (3.55–4.15)	8.5 (8.3–8.8)	<.001
Cefalexin	J01DB01	2112 (13.0)	10 285 (1.9)	3.05 (2.86–3.25)	8.8 (8.5–9.0)	<.001	1297 (11.7)	8835 (1.6)	2.96 (2.75–3.20)	7.7 (7.4–8.0)	<.001
Pantoprazole	A02BC02	5152 (31.8)	76 620 (13.9)	1.33 (1.27–1.39)	7.9 (6.8–8.9)	<.001	3213 (28.9)	72 730 (13.5)	1.10 (1.05–1.16)	2.7 (1.3–4.0)	.04
Ferrous fumarate	B03AA02	2153 (13.3)	17 291 (3.1)	1.71 (1.61–1.82)	5.5 (5.0–6.0)	<.001	1243 (11.2)	15 956 (3.0)	1.54 (1.43–1.66)	3.9 (3.3–4.4)	<.001
Furosemide	C03CA01	3676 (22.7)	32 906 (6.0)	1.31 (1.24–1.38)	5.4 (4.3–6.3)	<.001	2101 (18.9)	30 178 (5.6)	1.05 (.98–1.12)	.8 (−.4 to 2.0)	>.99
Codeine-acetaminophen	N02AJ06	1526 (9.4)	18 161 (3.3)	1.52 (1.42–1.62)	3.2 (2.8–3.6)	<.001	961 (8.6)	16 730 (3.1)	1.39 (1.28–1.51)	2.4 (1.9–2.9)	<.001
Loperamide	A07DA03	756 (4.7)	1736 (0.3)	3.54 (3.15–3.99)	3.4 (3.2–3.5)	<.001	362 (3.3)	1553 (0.3)	2.69 (2.31–3.13)	2.0 (1.8–2.2)	<.001
Amoxicillin	J01CA04	1266 (7.8)	19 017 (3.5)	1.53 (1.42–1.64)	2.7 (2.3–3.1)	<.001	908 (8.2)	17 390 (3.2)	1.43 (1.32–1.55)	2.4 (2.0–2.9)	<.001
Mesalazine	A07EC02	396 (2.4)	1935 (0.4)	5.33 (4.64–6.12)	2.0 (1.9–2.0)	<.001	294 (2.6)	1868 (0.3)	4.91 (4.21–5.71)	2.1 (2.0–2.2)	<.001
Prednisone	H02AB07	1614 (10.0)	12 768 (2.3)	1.34 (1.24–1.44)	2.5 (1.9–3.1)	<.001	927 (8.3)	11 413 (2.1)	1.17 (1.07–1.28)	1.2 (.5–1.8)	.23
Allopurinol	M04AA01	1286 (7.9)	19 469 (3.5)	1.39 (1.29–1.49)	2.2 (1.8–2.6)	<.001	797 (7.2)	18 763 (3.5)	1.25 (1.15–1.37)	1.4 (.9–1.9)	<.001
Ondansetron	A04AA01	520 (3.2)	959 (0.2)	2.82 (2.37–3.35)	2.1 (1.9–2.3)	<.001	227 (2.0)	766 (0.1)	1.83 (1.46–2.29)	.9 (.6–1.2)	<.001
Hydromorphone	N02AA03	1888 (11.7)	11 469 (2.1)	1.26 (1.17–1.35)	2.4 (1.7–3.0)	<.001	886 (8.0)	9052 (1.7)	1.08 (.99–1.19)	.6 (−.1 to 1.3)	>.99
PAR ≤−2%											
Metformin	A10BA02	1651 (10.2)	58 758 (10.7)	.67 (.63–.72)	−5.0 (−6.1 to −4.0)	<.001	1042 (9.4)	56 960 (10.6)	.58 (.54–.63)	−6.7 (−8.0 to −5.5)	<.001
Rosuvastatin	C10AA07	4312 (26.6)	137 349 (25.0)	1.02 (.97–1.07)	.5 (−.7 to 1.7)	>.99	2902 (26.1)	133 829 (24.8)	.74 (.71–.78)	−9.0 (−10.9 to −7.3)	<.001
Amlodipine	C08CA01	3692 (22.8)	95 025 (17.3)	.95 (.91–1.00)	−1.2 (−2.3 to −.1)	>.99	2392 (21.5)	91 793 (17.0)	.78 (.74–.83)	−5.9 (−7.4 to −4.5)	<.001
Senna glycosides	A06AB56	2000 (12.3)	21 745 (4.0)	.83 (.78–.89)	−2.5 (−3.5 to −1.5)	<.001	1084 (9.7)	19 362 (3.6)	.71 (.65–.77)	−4.0 (−5.2 to −2.9)	<.001
Atorvastatin	C10AA05	3731 (23.0)	95 032 (17.3)	1.06 (1.01–1.12)	1.4 (.3–2.4)	>.99	2418 (21.7)	92 156 (17.1)	.80 (.75–.84)	−5.6 (−7.2 to −4.1)	<.001
Rivaroxaban	B01AF01	796 (4.9)	16 165 (2.9)	.83 (.76–.90)	−1.0 (−1.6 to −.5)	.008	457 (4.1)	14 950 (2.8)	.64 (.57–.71)	−2.3 (−3.1 to −1.6)	<.001
Acetaminophen	N02BE01	2861 (17.7)	35 207 (6.4)	.98 (.92–1.04)	−.4 (−1.4 to .6)	>.99	1608 (14.4)	31 704 (5.9)	.84 (.78–.90)	−2.8 (−4.0 to −1.6)	<.001
Levothyroxine sodium	H03AA01	3269 (20.2)	75 654 (13.8)	1.03 (.98–1.07)	.5 (−.4 to 1.4)	>.99	2264 (20.3)	73 581 (13.6)	.85 (.81–.90)	−3.6 (−4.9 to −2.4)	<.001
Apixaban	B01AF02	1823 (11.3)	23 291 (4.2)	1.04 (.98–1.12)	.5 (−.3 to 1.2)	>.99	1092 (9.8)	21 682 (4.0)	.81 (.75–.88)	−2.3 (−3.3 to −1.3)	<.001

Abbreviations: ATC, anatomic therapeutic classification; CI, confidence interval; OR, odds ratio; PAR, population-attributable risk.

^a^The PAR represents the estimated reduction in cases if the given drug had not been received, expressed in terms of the proportion of cases. Estimates with PAR between −2 and 2 are in gray and are provided for comparison. Ordering is based on the average PAR between the primary and secondary analyses.

^b^ *P* values have been adjusted for multiple testing using the Bonferroni method.

Across the primary and secondary analyses, antibiotics were a major driver of drug-associated CDI risk. In the primary analysis, antibiotics represented 35% (51.9/147.0) of drug-associated PAR, compared with 53% (49.7/94.7) in the secondary analyses. In particular, 4C antibiotics (amoxicillin-clavulanate, fluoroquinolones, cephalosporins, and clindamycin) represented the vast majority of antibiotic-associated PAR (primary, 84% [43.4/51.9]; secondary, 85% [42.3/49.7]). This was mainly due to amoxicillin-clavulanate (PAR_primary_, 12.4 [95% confidence interval (CI), 12.2–12.5]), clindamycin (8.4 [8.3–8.4]), ciprofloxacin (9.6 [9.3–9.8]), and cephalexin (8.8 [8.5–9.0]), which had the highest PARs in both primary and secondary analyses. Outside of 4C antibiotics, amoxicillin, nitrofurantoin, and trimethoprim-sulfamethoxazole had PARs >1%.

Beyond antibiotics, pantoprazole had a weak OR but nonetheless a high PAR in the primary analysis, which was notably lower in the secondary analysis (OR_primary_ and OR_secondary_, 1.33 [95% CI, 1.27–1.39] and 1.10 [1.05–1.16], respectively). ORs for other PPIs were comparable to that for pantoprazole. Oral iron supplementation with ferrous fumarate had moderate ORs in both primary and secondary analyses (OR_primary_ and OR_secondary_, 1.71 [95% CI, 1.61–1.82] and 1.54 [1.43–1.66]). The second most commonly used oral iron supplement, ferrous gluconate, was also associated with CDI risk (OR_primary_ and OR_secondary_, 1.56 [95% CI, 1.41–1.72] and 1.39 [1.23–1.57]). Furosemide was associated with CDI risk in the primary but not the secondary analysis (OR_primary_ and OR_secondary_, 1.31 [95% CI, 1.24–1.38] and 1.05 [.98–1.12]). The combination analgesic, codeine-acetaminophen, was associated with risk in the primary and secondary analyses (OR_primary_ and OR_secondary_, 1.52 [95% CI, 1.42–1.62] and 1.39 [1.28–1.51]).

In terms of protective associations, the antidiabetic agent metformin had a protective association in both the primary and secondary analyses (OR_primary_ and OR_secondary_, 0.67 [95% CI, .63–.72] and 0.58 [.54–.63]). The most common metformin combination, metformin-sitagliptin, also appeared protective (OR_primary_ and OR_secondary_, 0.71 [95% CI, .63–.80] and 0.57 [.50–.67]), but the antidiabetic sitagliptin alone did not (1.31 [1.18–1.45] and 1.14 [1.00–1.30]). Protective associations were observed for many more drugs in the secondary than in the primary analyses; these included the commonly prescribed cardiac medications rosuvastatin (OR_primary_ and OR_secondary_, 1.02 [95% CI, .97–1.07] and 0.74 [.71–.78]), atorvastatin (1.06 [1.01–1.12] and 0.80 [.75–.84]), and amlodipine (0.95 [.91–1.00] and 0.78 [.74–.83]).

### Sensitivity Analyses

**Figure 3.**
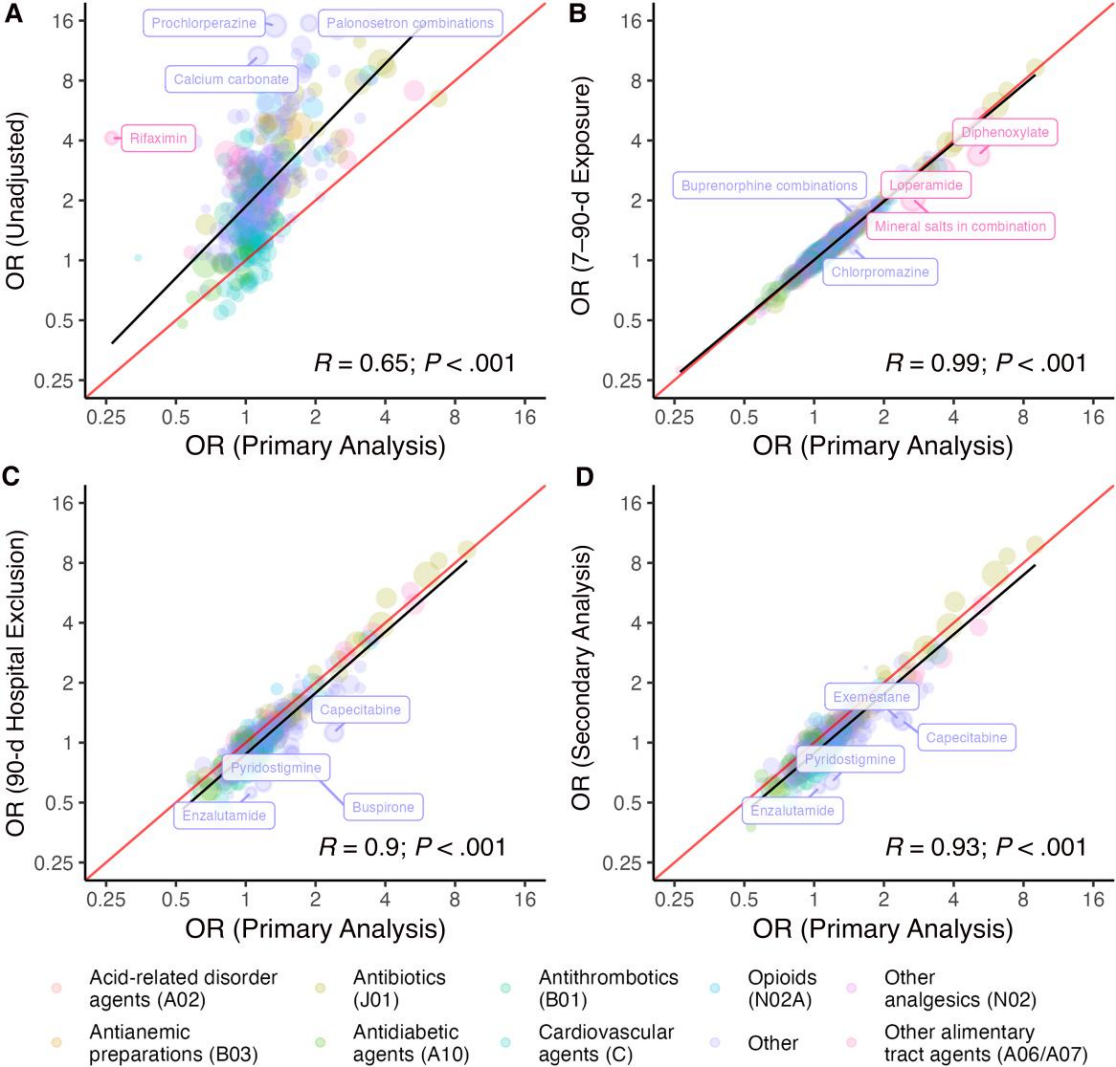
Comparison of primary adjusted analysis (x-axis) with unadjusted analysis (*A*), robust (7–90-day) exposure window (*B*), exclusion of hospitalization within prior 90 days (*C*), and secondary analysis (*D*). Indicated drugs within each panel had the largest absolute differences in log odds ratio (OR) between the 2 analysis approaches. *R* values represent Pearson correlation coefficients.

We systematically compared ORs obtained from our primary analysis, with ORs obtained from secondary analyses and sensitivity analyses (Figure 3). Compared to the primary analysis, almost all unadjusted estimates (SA1) were larger, and these were only moderately correlated with the primary analysis (Figure 3*A*; Pearson correlation *R* = 0.68), indicating strong impacts of confounding in the unadjusted estimates. Compared with the primary analysis, ORs based on exposures measured in the short window (SA2, not shown; *R* = 0.97) were larger in magnitude, while ORs based on exposures measured in the long window (SA3, not shown; *R* = 0.95) were smaller in magnitude. Compared with the primary analysis, ORs based on exposures measured in the 7–90-day window (SA4) were almost identical (Figure 3*B*; *R* = 0.99); however, as hypothesized, several antipropulsives (drugs that could be used to treat symptoms of CDI), including loperamide (OR_primary_ and OR_secondary_, 3.54 [95% CI, 3.15–3.99] and 2.69 [2.31–3.13], respectively) and diphenoxylate (5.12 [4.12–6.37] and 3.80 [2.86–5.05]) saw substantial reductions in the magnitude of association, suggesting protopathic bias in the primary analysis. Compared with the primary analysis, the analysis excluding patients with hospitalization in the prior 90 days (SA5) (Figure 3*C*; *R* = 0.94) and the secondary analysis (Figure 3*D*; *R* = 0.94), had attenuated associations.

### Meta-regression of Drug *in* Vitro Antimicrobial Activity Versus Drug CDI Infection Risk

**Figure 4.**
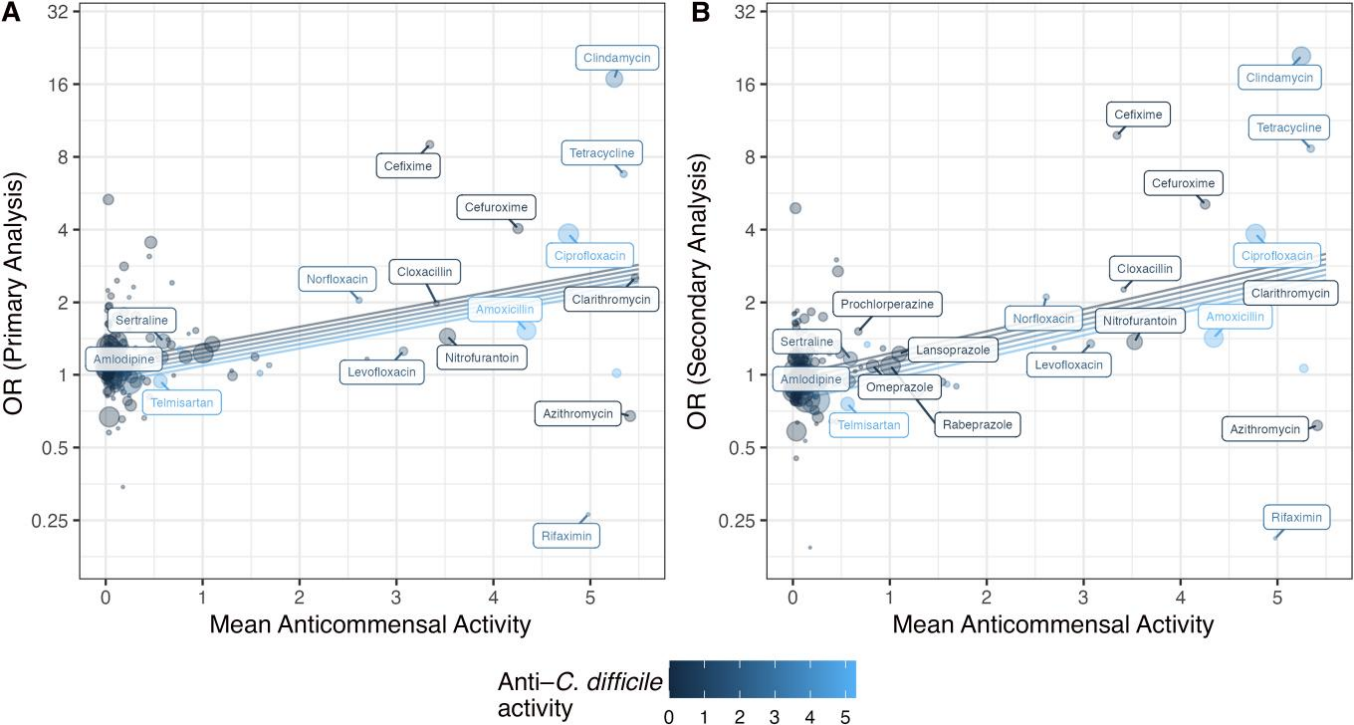
Meta-regression models of association between adjusted drug *Clostridioides difficile* infection odds ratios (ORs) (y-axes) and drug mean in vitro anticommensal activity (x-axes) and mean in vitro anti–*C. difficile* activity (0, *dark blue*; 5, *light blue*) in the primary (*A*; 182 drugs with antimicrobial activity data) and secondary (*B*; 179 drugs with antimicrobial activity data) analyses.

Antimicrobial activity information was available for 54% of the drugs included in the main analysis (primary analysis, 182 of 335; secondary analysis, 179 of 330). Among these drugs, we examined associations between in vitro anti–*C. difficile* activity and anticommensal activity and adjusted CDI ORs from our primary and secondary analyses (Figure 4 and Supplementary Table 3). In the primary analysis (1–90-day exposure window and 3–30-day hospitalization exclusion), each 1-point increase in anticommensal activity was associated with a 1.18-fold increase in the drug OR (95% CI, 1.11–1.26-fold), while each 1-point increase in anti–*C. difficile* activity was associated with a nonsignificant 0.96-fold decrease in drug OR (.89–1.04). In the secondary analysis (7–90-day drug exposure window and 1–90-day hospitalization exclusion), associations were stronger; each 1-point increase in anticommensal activity was associated with a 1.23-fold increase in the drug OR (95% CI, 1.15–1.32-fold), while each 1-point increase in anti–*C. difficile* activity was associated with a nonsignificant 0.95-fold decrease in the drug OR (.88–1.03-fold).

## DISCUSSION

This pharmacopeia-wide association study of drug-associated CDI risks confirmed that antibiotics, in particular 4C antibiotics (amoxicillin-clavulanate, fluoroquinolones, cephalosporins, and clindamycin) contribute disproportionately to the burden of CDI. In addition, several nonantimicrobial drugs, particularly ferrous fumarate, and codeine-acetaminophen, were associated with increased CDI incidence. Our secondary analysis also identified metformin, several Hydroxymethylglutaryl-CoA reductase inhibitors (statins), and amlodipine, as associated with reduced CDI incidence.

This study identified that 4C antibiotics remain some of the main drivers of CDI rates, despite stewardship efforts to curtail the overuse of these agents [17]. These 4C antibiotics were the target of national antimicrobial stewardship efforts in Scotland, and the strategy led to large reductions in CDI incidence [18]. Our study suggests that efforts to specifically reduce the use of 4C agents in outpatients may be beneficial in reducing CDI risk. When prescribing is indicated, alternative lower-risk and narrower-spectrum agents are usually available (eg, preferred prescribing of amoxicillin over amoxicillin-clavulanate or clindamycin where appropriate, preferred use of nitrofurantoin over ciprofloxacin or cephalexin when prescribing for uncomplicated cystitis).

We are not aware of any prior studies in human populations examining CDI risks associated with iron supplementation. Several mechanistic studies have shown that *C. difficile* bacteria have sophisticated approaches to iron homeostasis [19, 20]; in vitro data show that iron can increase *C. difficile* growth and toxin production [21]. Caution may be warranted with iron supplementation in patients with CDI risk factors, such as recent hospital exposure or concurrent 4C antibiotic exposure, and in patients being treated for CDI.

Our findings regarding PPIs are mixed. Meta-analyses have noted substantial interstudy heterogeneity in PPI estimates [22]. Our analyses provide some insight into potential drivers of interstudy heterogeneity. PPIs are frequently initiated during hospitalization and patients often continue receiving them after discharge [23, 24]. As such, residual confounding due to recent healthcare exposure, an extremely strong risk factor [25], could be an explanation for interstudy heterogeneity as well as the mixed findings within our study.

Prior studies have identified metformin use among diabetic patients as protective against CDI (OR, 0.58 [95% CI .37–.93] [26] and 0.48 [95%CI .45–0.51, 27]). Our study identified a similar magnitude of protective benefit of metformin. Several mechanisms for this association are possible; in particular, metformin may increase production of short-chain fatty acids, including metal-binding proteins and butyrate in gut bacteria [28]. Metformin could have a role in the prevention and treatment of CDI, either as a prophylactic agent or as an adjuvant, though randomized evidence is needed.

A meta-analysis indicated that statins are likely protective against CDI (OR, 0.73 [95% CI, .60–.88]) [29], and our secondary analyses identified a similar magnitude of association (OR for rosuvastatin, 0.74 [95% CI, .71–.78]; OR for atorvastatin, 0.80 [.75–.84]). We also observed that the calcium channel blocker amlodipine had a protective association. Our model of antimicrobial activity indicated that amlodipine had substantial anti–*C. difficile* activity without strong activity against gut commensal bacteria, suggesting antimicrobial activity against *C. difficile* bacteria as a potential mechanism.

Our study provides 335 adjusted estimates of drug-associated CDI risk, including multiple sensitivity analyses providing insights on duration of risks, confounding, and reverse causation. One prior study examined 290 medications and their association with CDI risk [27]; a strength relative to this prior study is that we provide a ranking of drugs based on PAR, boosting the clinical and stewardship relevance of our findings. We also examined the association between the anticommensal activity of drugs and CDI risk, leveraging comprehensive estimates of in vitro activity [4]. Increased in vitro activity against gut commensals was associated with increased CDI risk, and these analyses explained 19%–27% of the variability in drug-associated risk. The moderate strength of the association could indicate that other mechanisms not captured by in vitro models also drive CDI risk (eg, impacts of PPIs on stomach pH [30] and impacts of opioids on gut motility [31]). However, improvements to in vitro measurement of antimicrobial activity are also possible, such as through use of multiple more representative strains for measurement of anti–*C. difficile* activity [32, 33] or through use of a synthetic microbiome community for in vitro measurement of anticommensal activity [34].

Our study is subject to a number of limitations related to (1) residual confounding, (2) protopathic bias, (3) multiple testing, and (4) misclassification of the outcome. First, residual confounding, including confounding by indication, may occur in any observational study due to incomplete adjustment for potential confounders (eg, over-the-counter medications or longer or shorter than average medication duration); however, our study adjusted for more potential confounders than prior studies, including patient comorbid conditions, healthcare exposure history, and >300 medication coexposures. Second, protopathic bias may have occurred in our study when a drug was used to treat a symptom of CDI, before receipt of a positive *C. difficile* result and initiation of a CDI treatment agent. This could have occurred with antidiarrheals such as loperamide and diphenoxylate, which were identified as a strong risk factors for CDI in our primary analysis. Our secondary analysis, which combatted protopathic bias by not including exposures in a 1–6-day window before CDI (ie, using a 7–90-day window instead of a 1–90-day window), demonstrated substantially diminished risk estimates for loperamide and diphenoxylate. Third, we adjusted our *P* value estimates using the Bonferroni method to account for multiple testing. Fourth, our outcome was based on a nucleic acid amplification test– or enzyme immunoassay–positive result, a CDI *International Classification of Diseases* code, or identification of CDI antimicrobial therapy—a high-sensitivity but potentially low-specificity approach. Finally, while our model accounted for confounding due to coprescribing, we did not further explore statistical interactions between drugs.

This study identifies important associations between antibiotic and nonantibiotic drug exposures and CDI risk, provides insights into the etiology of CDI while demonstrating the need for more predictive microbiome disruption measures, and signals some potential avenues for stewardship efforts and repurposing of commonly prescribed nonantibiotic drugs in the fight against *C. difficile* and antimicrobial resistance.

## Supplementary Material

jiag001_Supplementary_Data

## Supplementary Data

Supplementary materials are available at *The Journal of Infectious Diseases* online (http://jid.oxfordjournals.org/). Supplementary materials consist of data provided by the author that are published to benefit the reader. The posted materials are not copyedited. The contents of all supplementary data are the sole responsibility of the authors. Questions or messages regarding errors should be addressed to the author.
